# A novel high accuracy model for automatic surgical workflow recognition using artificial intelligence in laparoscopic totally extraperitoneal inguinal hernia repair (TEP)

**DOI:** 10.1007/s00464-023-10375-5

**Published:** 2023-08-25

**Authors:** Monica Ortenzi, Judith Rapoport Ferman, Alenka Antolin, Omri Bar, Maya Zohar, Ori Perry, Dotan Asselmann, Tamir Wolf

**Affiliations:** 1 Theator Inc., Palo Alto, CA USA; 2 Department of General and Emergency Surgery, Polytechnic University of Marche, Ancona, Italy

**Keywords:** Surgical intelligence, Minimally invasive surgery, AI, TEP, Inguinal hernia repair

## Abstract

**Introduction:**

Artificial intelligence and computer vision are revolutionizing the way we perceive video analysis in minimally invasive surgery. This emerging technology has increasingly been leveraged successfully for video segmentation, documentation, education, and formative assessment. New, sophisticated platforms allow pre-determined segments chosen by surgeons to be automatically presented without the need to review entire videos. This study aimed to validate and demonstrate the accuracy of the first reported AI-based computer vision algorithm that automatically recognizes surgical steps in videos of totally extraperitoneal (TEP) inguinal hernia repair.

**Methods:**

Videos of TEP procedures were manually labeled by a team of annotators trained to identify and label surgical workflow according to six major steps. For bilateral hernias, an additional change of focus step was also included. The videos were then used to train a computer vision AI algorithm. Performance accuracy was assessed in comparison to the manual annotations.

**Results:**

A total of 619 full-length TEP videos were analyzed: 371 were used to train the model, 93 for internal validation, and the remaining 155 as a test set to evaluate algorithm accuracy. The overall accuracy for the complete procedure was 88.8%. Per-step accuracy reached the highest value for the hernia sac reduction step (94.3%) and the lowest for the preperitoneal dissection step (72.2%).

**Conclusions:**

These results indicate that the novel AI model was able to provide fully automated video analysis with a high accuracy level. High-accuracy models leveraging AI to enable automation of surgical video analysis allow us to identify and monitor surgical performance, providing mathematical metrics that can be stored, evaluated, and compared. As such, the proposed model is capable of enabling data-driven insights to improve surgical quality and demonstrate best practices in TEP procedures.

**Graphical abstract:**

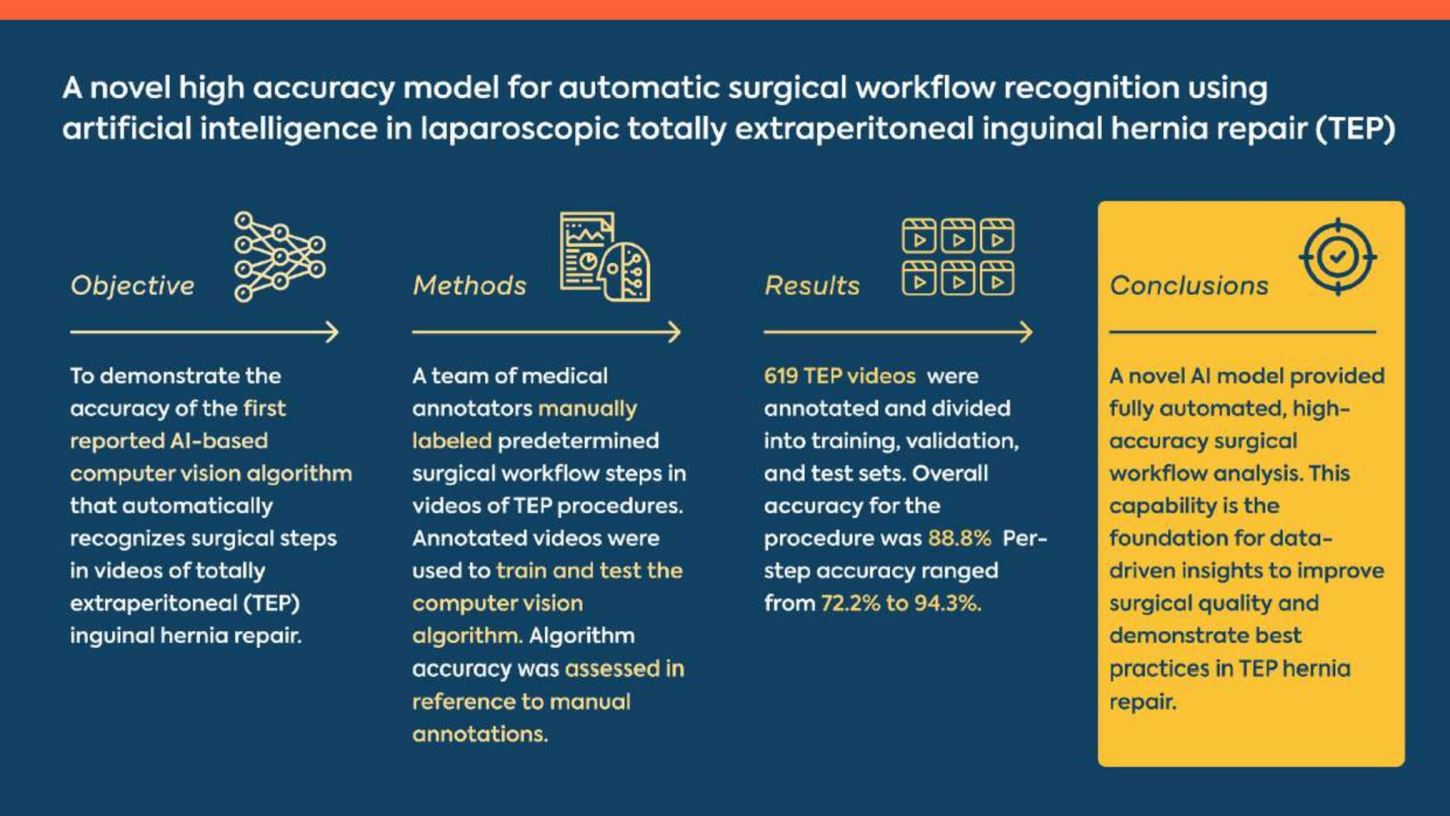

Artificial intelligence (AI) is increasingly being adopted in several medical specialties. It has proven to be an effective supporting tool in various fields and at different levels of healthcare management, from diagnosis to treatment and follow up [1, 2]. Since its first introduction, surgery mediated by a video interface has evolved from a hardly conceivable concept to an efficient tool that can be harnessed to achieve various goals [3]. Surgical videos document intraoperative events in a more objective and detailed manner than do operative notes alone, enabling accurate review of surgical performance and, eventually, correlation to patient outcomes [4–6]. As such, groups in both industry and academia are exploring ways to efficiently record, collect, store, and analyze surgical videos, with the ultimate goal of providing intraoperative decision support to improve surgical quality and, consequently, outcomes [7, 8].

Video analysis per se is an important tool that allows surgeons to gain valuable insights into their surgical practice. However, full video review is incredibly time-consuming, and surgeons rarely have the time and technological tools to perform it routinely. As a result, significant amounts of actionable data are lost [9]. The introduction of AI presents an opportunity to overcome these gaps in video utilization and enables the surgical field to maintain continuous technological advancement.

Surgical Intelligence platforms available today integrate AI and computer vision tools, allowing automation of surgical video capture and analysis. This transforms long, unedited surgical videos into segmented, user-friendly videos that present data regarding key surgical steps, important intraoperative events, and efficiency metrics. Eventually, the goal will be for real-time information regarding ongoing operations to enable surgical workflow optimization [10]. These technological capabilities hold great promise for a variety of clinical and quality assurance applications, from education and training to individual analytics and insights, alongside departmental identification of blind spots, efficiency metrics, and more [11].

High-accuracy detection of surgical steps provides an important foundation for these capabilities and has been studied recently across several surgical specialties [12–14] and procedures [15–17]. Segmenting procedural video into key steps allows for easier navigation of the video, enables the presentation of additional data like intraoperative adverse events and safety achievements, and provides valuable information for workflow optimization. Despite ongoing advancements in this field [17], prior step recognition models have been based on limited datasets. Even when they demonstrate good accuracy levels, these models lack generalizability [18] and are less likely to capture the variability and complexity of step detection in procedures performed by different surgeons, using varied techniques, at facilities of varying professional levels.

Inguinal hernia repair can be considered one of the most relevant fields to test and employ an AI video analysis model, for two main reasons. First, groin hernia repair is one of the most commonly performed operations in general surgery [19, 20]. Second, minimally invasive techniques have been successfully incorporated into inguinal hernia surgery and are used more commonly due to their proven advantage in comparison to open techniques, with fewer postoperative complications and faster recovery times [21, 22]. Still, the choice of surgical technique for groin hernia repair remains controversial due to the technical skills required to perform minimally invasive, as compared to conventional, techniques [19, 23]. Previous studies on automated step detection in hernia surgery focused mainly on transabdominal preperitoneal repair (TAPP) [24, 25]. The aim of this study was to develop, test, and demonstrate the accuracy of a novel computer vision algorithm for automated surgical step detection of laparoscopic totally extraperitoneal (TEP) inguinal hernia repair.

## Materials and methods

### Dataset

The dataset for this study comprised 619 TEP videos collected between October 25, 2019, and December 13, 2022. A Surgical Intelligence platform (Theator Inc., Palo Alto, CA) was used to automatically capture the videos during surgery and to de-identify and upload procedures to a secure cloud infrastructure. Data was then analyzed retrospectively. Of the videos, 602 were curated from 3 medical centers and 17 from other sources, with a total of 85 expert surgeons participating. The final set of 619 was reached after excluding videos documenting TAPP procedures as well as videos in which the recording quality and/or the number of steps documented were insufficient for analysis.

Surgical video data were organized and annotated (see “Annotation process” section) using a dedicated platform. Videos were divided into two groups based on the type of hernia repair they documented (unilateral or bilateral). Total duration of the procedure was defined from the first camera insertion to the end of the final inspection and final camera extraction. The operative procedure was standard and comprised a succession of predefined steps, from access to the preperitoneal space to its closure after mesh placement [26, 27]. Type of access to the preperitoneal space, either with the aid of a balloon dissection or with direct access, without the use of a space maker, was documented. Mesh was systematically used in this series and fixed over the myopectineal orifice (MPO) [3, 28, 29]. All videos were obtained following IRB approval.

### Annotation process

In this context, the term annotation refers to the process of manually marking the start and end points of each procedural step. The general annotation process was based on the process developed and validated in a previous study [30].

A team of annotators previously trained with respect to the TEP procedure manually annotated all the videos. To maintain high standards, the process included three levels of validation. First, each video was randomly assigned to a trained annotator who reviewed it and manually assigned each second of the procedure to one of the predefined surgical steps. This was followed by review by a second trained annotator (i.e., validator), who validated the labels with the aim of reducing manual errors and enforcing an aligned annotation process. In cases with unclear workflow or non-typical events, a second validation was conducted by a general surgeon with experience in laparoscopic hernia repair procedures. The manually annotated videos were subsequently used to train and test the computer vision AI algorithm.

### Step definitions

The annotation system was based on the recognition of 6 major procedural steps: (1) balloon dissection, (2) access to the preperitoneal space, (3) preperitoneal dissection, (4) hernia sac reduction, (5) mesh placement, and (6) mesh fixation. For bilateral hernias, an additional step, change of focus, designated the switching of sides during the procedure. The defined steps represent a complete framework for TEP, but not all documented procedures include them all, and they are independent of one another and of chronological order. As such, when a step did not occur, it did not interfere with recognition of the other steps.

The term ‘out of body’ indicates video sequences during which the camera was extracted from the optical trocar, for example, to clean the camera or to introduce the mesh through wider access to the abdomen [27]. The start and end points of each step were defined based on technical surgical details (Table 1). A guiding rule for the entire annotation process was, ‘One step ends when the next step starts,’ such that every second of video was labeled as one and only one of the steps. Rules for step recognition were set ahead of the annotation process. Standardized definitions were produced based on pre-existing recommendations, international guidelines, and joint consultation with expert surgeons and AI engineers, taking into account clinically relevant and algorithmically meaningful considerations [26, 27].

**Table 1:** Procedural steps with visual triggers and associated surgical actions

Procedural step	Trigger	Surgical actions
Balloon dissection	First view of the dissection balloon	Begin removing peritoneum from adhered fat tissue
Extraperitoneal access (trocars)	Insertion of the first operative trocar	Insertion of laparoscopic instruments (e.g., trocars, dissectors)
Preperitoneal dissection	Beginning of the dissection of the preperitoneal space	Visualizing the pubic bone/Cooper’s ligament, inferior epigastric vessels, creating a dissection space surrounding the hernia sac
Hernia and sac reduction	First pulling of the hernia sac	Completing reduction of the sac
Mesh placement	First view of the inserted mesh	Passing the mesh through the patient’s abdomen and placing it in a desired position
Mesh fixation	First view of the tacker or suture (or glue)	Positioning the tacker/suture to fix the mesh
Change of focus (left to right, right to left)	Switch of camera focus contralaterally	Beginning preperitoneal dissection on the opposite site

### Algorithm architecture

Videos in this study were all processed with the same AI model, which comprised several parts, as follows:

#### Video preprocessing

Raw video files were processed using FFmpeg. First, audio was removed and videos were encoded with libx264, using 25 frames per second. Then, video width was scaled to 480, and height was set to maintain the aspect ratio of the original video. We only considered video segments from initial insertion to final extraction of the scope, so video footage before or after these timepoints was discarded. In addition, non-relevant frames, such as out-of-body frames recognized during the surgery itself, were de-identified by an image-blurring algorithm to maintain patient privacy and surgical team confidentiality [31].

#### Step recognition model

The method utilized in this study, as previously described in Bar et al. [18], consisted of two key modules: (1) a feature extraction model that produced a mathematical representation of each second in the procedural video and (2) a temporal model that learned to predict surgical steps based on the sequence of features.

Generally, videos could be processed either in an end-to-end manner (i.e., frame-by-frame) or as short clips. Often, video-based methods use short clips, which are typically suitable when analyzing short videos. However, surgical videos can be hours long, and in cases like these, end-to-end processing achieves better performance and accuracy while also surveying the entire surgical procedure. In this study, we employed a Video Transformer Network (VTN) [32] as a feature extraction model. VTN processes videos as a sequence of images (video frames) and uses attention modules [33] to focus on specific important cues in the video. These temporal-visual features enable differentiation of important actions that take place in the video (such as the entry of a surgical tool into the visual field) from idle, prolonged segments (such as minutes-long adhesiolysis). When provided with surgical step annotations during training, our model learned to focus on the specific transitions necessary for accurate step recognition.

Previous studies have demonstrated that transfer learning can be utilized for step recognition in several general surgery procedures and that it improves algorithm performance compared to traditional approaches [32]. In this study, we applied transfer learning by using a model that had been pre-trained on multiple general surgery procedures (e.g., cholecystectomy, appendectomy, sleeve gastrectomy) and was then adjusted using the TEP videos. Model adaptation to TEP steps was accomplished with a temporal model based on a Long Short-Term Memory (LSTM) network [34]. The LSTM network processes long sequences, taking into account the current second representation as well as maintaining a “memory” of previous relevant information that contributes to the model's final predictions.

### Statistical analysis

Performance accuracy, defined as the accuracy of the AI algorithm in automatically recognizing the different steps, was assessed by comparison to the human annotations. Per-second accuracy was used to evaluate the model’s performance, which was calculated by comparing the manual annotation (ground truth) with the model’s step prediction for each second. Accuracy was defined as the ratio between the sum of correct predictions and the overall number of seconds. Mean-unweighted accuracy was defined as the average of the individual class accuracies. An error analysis was also performed by identifying the videos with the lowest accuracy in the test set and reviewing them individually. Continuous data were analyzed using the Mann–Whitney *U* test to examine the differences between unilateral and bilateral repairs when appropriate. A *p* value of < 0.05 was considered to be indicative of statistical significance.

## Results

A total of 619 full-length TEP videos were analyzed: 371 were used to train the model, 93 for internal validation, and the remaining 155 as a test set, to evaluate the algorithm accuracy. Among the included cases, 270 were unilateral inguinal repairs (166 training, 46 validation, 58 test) and 349 were bilateral (205 training, 47 validation, 97 test). The mean procedure duration was 37.5 min. The average duration of bilateral procedures was 43 min (std = 21 min, *n* = 349). The average duration of unilateral procedures was 29 min (std = 17 min, *n* = 270). There was a statistically significant difference in duration between unilateral and bilateral procedures (*p* < 0.001). A dissecting balloon was used in 352 cases (57%) [23].

**Fig. 1:**
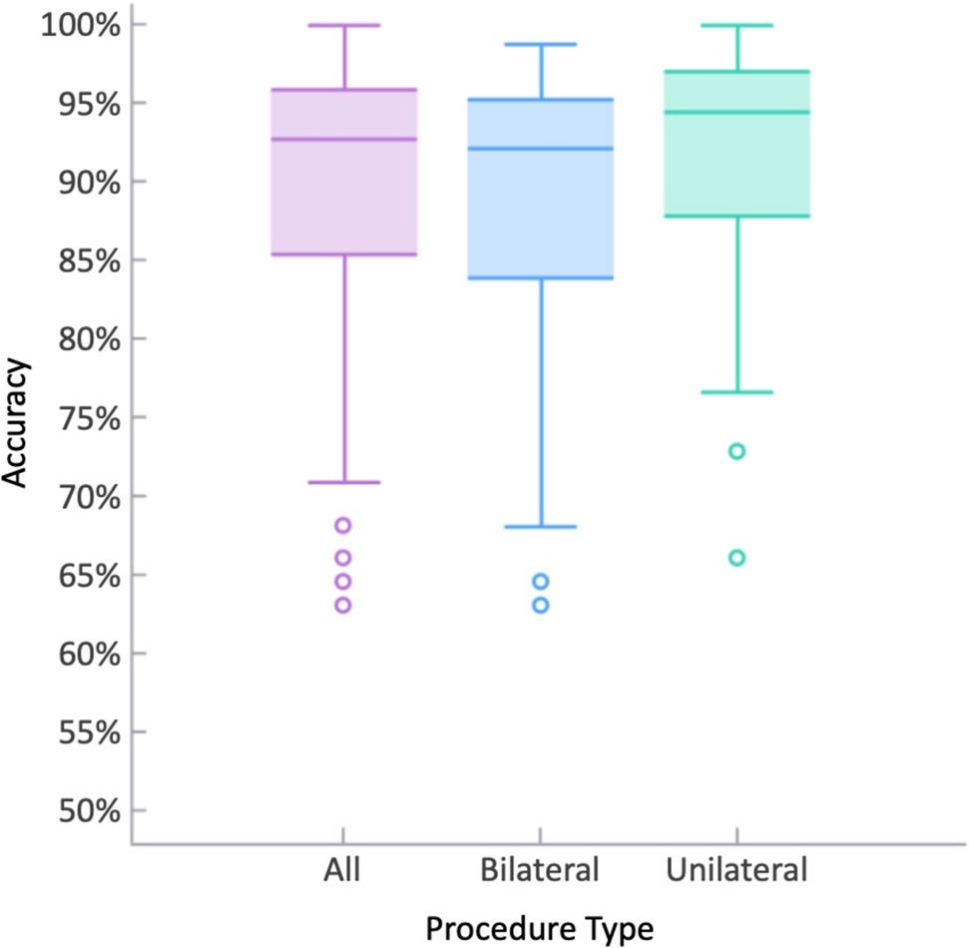
Box plots of the accuracy of the test set, with the *x*-axis indicating procedure type (all, bilateral, unilateral) and the *y*-axis indicating the accuracy of the model, with respect to manual annotations. Horizontal lines in the box and whisker plots indicate the 25th, 50th, 75th, and 100th percentiles. Dots represent outlying cases with the lowest accuracies

For unilateral repairs, the mean-unweighted accuracy was 89.16% and the overall accuracy was 89.59%, while for bilateral repairs the mean-unweighted accuracy was 83.17% and the overall accuracy was 88.45% (*p* = 0.0394; see Fig. 1).

Mesh placement was not performed in eight procedures (1.3%). When mesh placement was performed, it was not fixated in 36 procedures (5.81%) and was fixated in 575 procedures (92.9%). In 566 procedures (98%) fixation was accomplished with tackers, in 5 (0.87%) with glue, and in 4 (0.7%) with both tackers and glue. Per-step accuracy was highest for the hernia sac reduction step (94.3%) and lowest for the preperitoneal dissection step (73%), which was mostly confused with the hernia sac reduction step (25%).

**Fig. 2:**
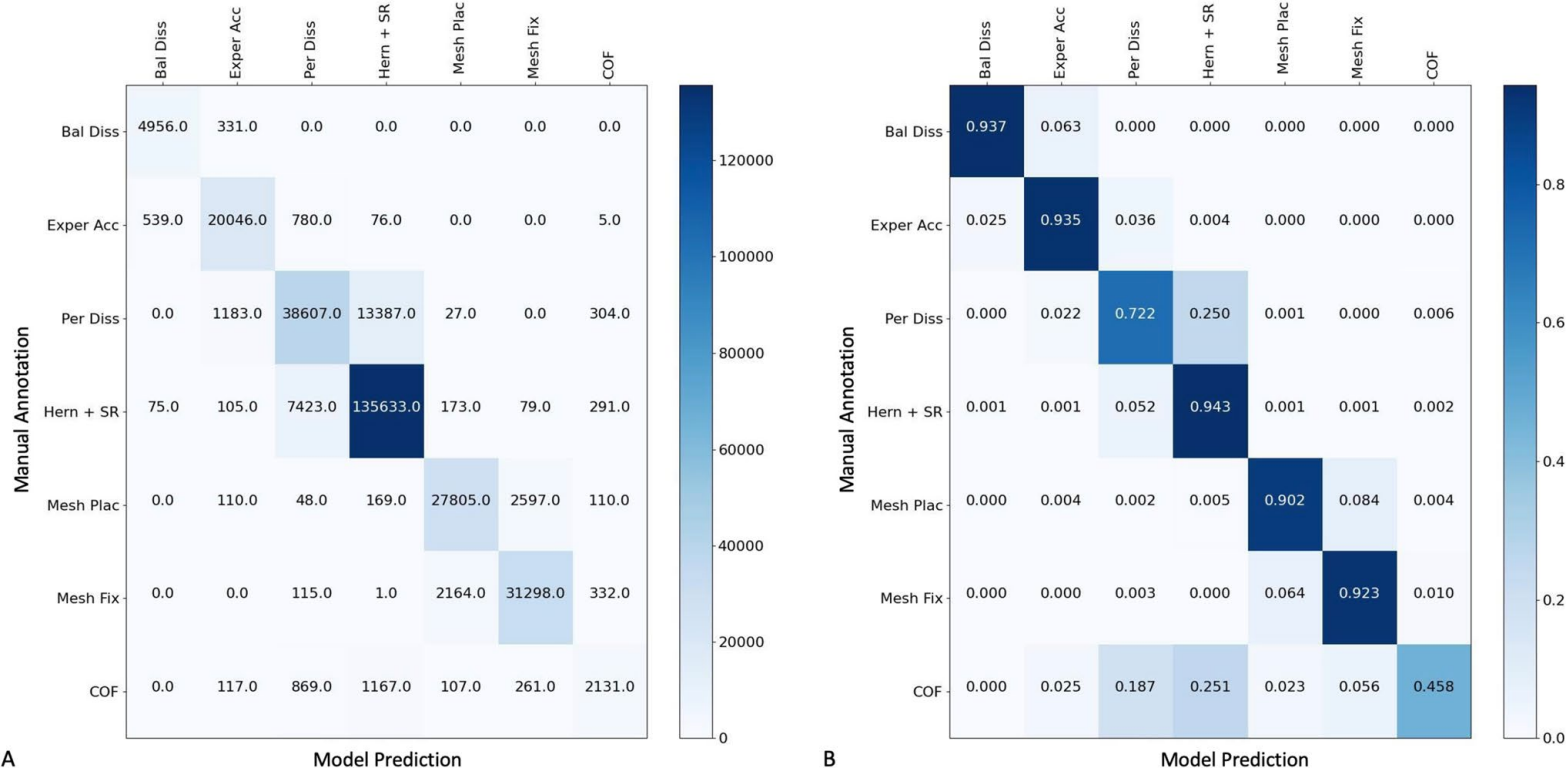
**A** Confusion matrix and **B** NCM presenting per-step accuracy of the automated step detection algorithm in reference to manual annotation in TEP hernia repairs. The matrices indicate the level of concordance between labels predicted by the model (*x*-axis) and those identified by human annotators (*y*-axis). Values on the diagonal represent the proportion of time points where the detection was accurate (in seconds in **A** and percentages in **B**, also called the true positive rate) for each step. Values not on the diagonal represent the degree of confusion by indicating the proportion of time points in which the model incorrectly labeled one step as another. For example, for the “Preperitoneal Dissection” step, the model and human annotations had an agreement rate of 72.2%. The relatively high values along the diagonal show that the model is highly capable in automatically detecting the TEP steps. *Bal Diss* balloon dissection, *Exper Acc* extraperitoneal access (trocars), *Per Diss* preperitoneal dissection, *Hern + SR*  hernia and sac reduction, *Mesh Plac* mesh placement, *Mesh Fix* mesh fixation, *COF* change of focus

Figure 2A shows the confusion matrix (CM) and Fig. 2B the normalized confusion matrix (NCM) for the model, to demonstrate the model’s performance. Rows correspond to annotated steps (manual annotations, i.e., ground truth) and columns correspond to steps recognized by the model. Values on the diagonal represent the proportion of time points (seconds in CM) for which the model detection was correct (also called the true positive rate [3] in NCM) for each step. Values not on the diagonal represent the proportion of time points for which the model detection was incorrect. The model showed true positive rates (diagonal) ranging from 94.3% (for sac reduction) to 72.2% (for preperitoneal dissection).

**Fig. 3:**
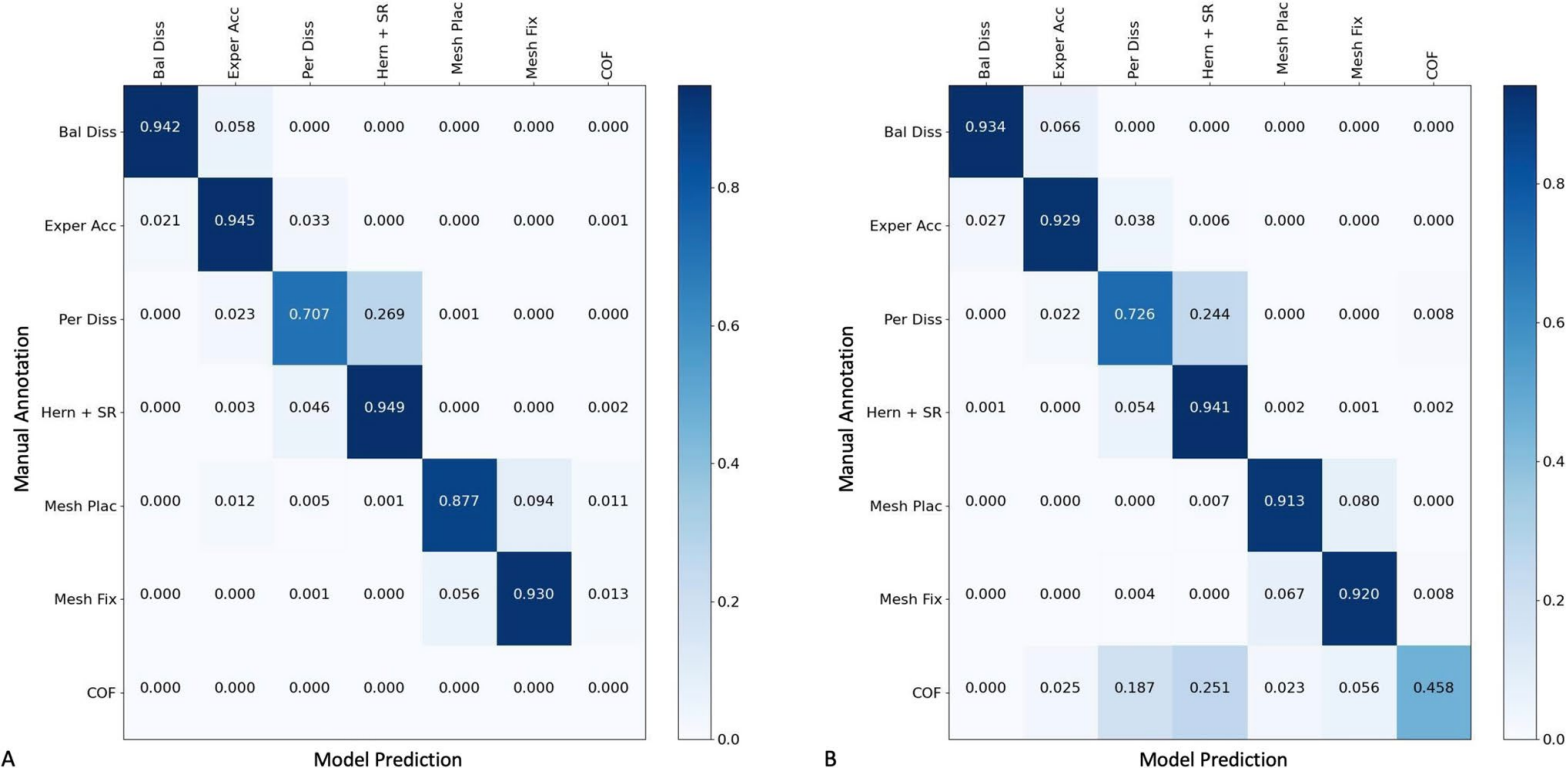
Normalized confusion matrix presenting per-step accuracy of the automated step detection algorithm in reference to manual annotation in unilateral (**A**) and bilateral (**B**) TEP hernia repairs. The NCM indicates the level of concordance between labels predicted by the model (*x*-axis) and those identified by human annotators (*y*-axis). Values on the diagonal represent the proportion of time points where the detection was accurate for each step. Values not on the diagonal represent the degree of confusion by indicating the proportion of time points in which the model incorrectly labeled one step as another. For example, for the “Preperitoneal Dissection” step, the model and human annotations had agreement rates of 70.7% and 72.6% for unilateral and bilateral procedures, respectively. *Bal Diss* balloon dissection, *Exper Acc* extraperitoneal access (trocars), *Per Diss* preperitoneal dissection, *Hern + SR* hernia and sac reduction, *Mesh Plac* mesh placement, *Mesh Fix* mesh fixation, *COF* change of focus

Figure 3 shows the NCM for unilateral (A) and bilateral repairs (B). The model shows true positive rates (diagonal) ranging from 94.9% (hernia sac reduction) to 70.7% (preperitoneal dissection) in unilateral repairs and from 94.1% (hernia and sac reduction) to 45.8% (change of focus) in bilateral repairs. Change of focus was mostly confused with preperitoneal dissection (25.1%).

### Error analysis

Of the 155 videos in the test set, those with the lowest accuracy were identified and reviewed. In the 97 bilateral repair videos, accuracy was below 70% in two videos (*n* = 2, 2%), 70–75% in 3 videos (*n* = 3, 3%), and 75–80% in nine videos (*n* = 9, 9.2%).

In the 58 unilateral repair videos, accuracy was below 70% in 1 video (*n* = 1, 1.7%), 70–75% in one video (*n* = 1, 1.7%), and 75–80% in 2 videos (*n* = 2, 3.4%).

Two main common features were identified in the procedures with the lowest accuracy levels. First, many had an unusual or rare feature/occurrence (different type of access, large sac tear making the dissection intraperitoneal, etc.). For example, a case with 68% accuracy was a single port procedure requiring an unconventional set up. The second common feature associated with low accuracy was the presence of a very subtle transition between hernia reduction and preperitoneal dissection. Interchanging of these two steps during the course of the surgery might cause initial inaccuracies in step starting times.

## Discussion

This study reports on the first AI-based automated recognition of surgical steps in videos of TEP inguinal hernia repair, reaching accuracy as high as 94.3% in recognition of key surgical steps. Our results show that per-step accuracy was highest for the hernia sac reduction step (94.3%). The lowest accuracy was observed for preperitoneal dissection (72.2%), which was mostly confused with hernia sac reduction (25%). This finding is not unexpected, since in the proposed TEP model, the hernia sac reduction step has a very clear trigger point corresponding to the first pulling of the sac. The preperitoneal dissection, however, usually starts in a more subtle way, as blunt dissection, which is less clearly detectable than sharp dissection. The temporal aspect may also contribute to the model’s confusion in this step. Namely, the hernia sac reduction step always takes place after a preperitoneal dissection is complete. The latter step is usually located after the access step, although an extra preperitoneal dissection might be necessary after the sac reduction, possibly disrupting the expected temporal sequence and creating another source of confusion.

An additional source of potential confusion was the transition between these steps, which can be unclear. As some dissection is always done together with the hernia reduction and some is done during trocar insertion, it is possible to mistakenly identify these steps, especially since dissection can occur more than once during the course of surgery.

Finally, the annotation process is “annotator-dependent” and inter-rater variability can be reflected in the annotator-model relationship. Small details are sometimes difficult to discern, even for an expert surgeon, and just as this could generate disagreement between two surgeons, it could be a source of disagreement between the model and the human annotator, hence lowering the accuracy of the model.

For unilateral repairs in this study, the mean-unweighted accuracy was 89.16% and the overall accuracy was 89.59%, while for bilateral repairs, the mean-unweighted accuracy was 83.17% and the overall accuracy was 88.45%. The task of surgical step recognition benefits from the fact that within the same procedure type, different procedures performed by different surgeons are still similarly structured. The procedural flow, the tools used, and the surrounding anatomical context remain similar no matter where the surgery is performed and who is performing it. Having said that, accurate step recognition is challenging due to the need to detect relatively small differences across time—a change in tools, a change in anatomic target, or a difference in how a tool is used.

By definition, AI is a mathematical approach that can be used to effectively reproduce tasks that normally require human intelligence [35]. To date, several approaches to step recognition using different types of machine learning have been performed by various researchers with an accuracy ranging from 69 to 86% [17, 18, 36–39]. Laparoscopic cholecystectomy was the first procedure on which surgical step recognition was studied, given its easy reproducibility, the existence of well-defined safety practices, and its ubiquitous presence in general surgery departments [39–41]. Later, Takekuchi et al. first proposed an AI model for step detection in TAPP, with an overall accuracy of 88.81% and 85.82% for unilateral and bilateral cases, respectively [22, 24].

The rationale for testing AI models on common procedures is twofold. The first is the ability to access large volumes of data in a relatively short period of time. The second is the need to give quick and useful feedback for the most commonly performed procedures, with the highest achievable accuracy, in order to justify the use of AI in clinical settings. Additionally, from a technological standpoint, this strategy allows us to explore and refine the models before applying them to more complex procedures [34].

Generalizability is one of the key characteristics contributing to the successful application of AI in the surgical field. It refers to the ability to accurately transfer the model’s knowledge to similar procedures, disregarding the many biases present when a variety of operating surgeons and venues are involved. Essentially, the ability to gather as much information as possible allows the model to adapt to new medical centers and surgeons without compromising accuracy [18]. To make this possible in the current study, transfer learning [35] was applied during the model’s training process, meaning that a model that had previously been trained on multiple general surgery procedures (e.g., cholecystectomy, appendectomy, sleeve gastrectomy) was adapted to be used for the TEP videos.

Another challenge in building AI models is the need for correctly labeled, representative data to drive the learning process. In the surgical field, the capture, labeling, and sharing of data are inherently more challenging than in other specialties [9]. As a result, most studies performed to date have been based on limited datasets [18] derived from single institutions [22, 24], and may therefore lack generalizability. The dataset used in the present study was composed of videos from a variety of venues and operating surgeons, providing significantly greater variability for training the model. In addition, the large-scale dataset and independent test set support the reliability and reproducibility of the results obtained.

The introduction of AI into surgery is an answer to the unmet need for standardization of surgical approaches. This need is well represented by quality improvement programs, which have been flourishing in recent years. One of these is the American College of Surgeons (ACS) National Surgical Quality Improvement Program (NSQIP) Quality Verification Program (QVP), a standards-based verification program designed to help sites improve quality across surgical departments [42, 43]. It operates under the motto, “you can’t improve quality if you can’t measure it,” which justifies effective implementation and integration of AI-supported surgical data science to analyze operative metrics in national quality programs.

The ultimate goal for AI, as for every other new technology that has been introduced into medical care, is to improve patient outcomes. Automated step detection facilitates surgical video navigation and review [39, 44]. Structuring the data (e.g., procedure steps and more) from surgical videos and connecting that information to preoperative and postoperative patient data and outcomes can offer novel insights when performed at scale. The step detection method presented in this study was conceived, amongst other use cases, to give surgeons the option of choosing whether to watch an entire procedure or to focus on a particular step. It also allows us to obtain analytics comparing performance across various procedures between a single surgeon and other surgeons from the same department, for example based on level of expertise and previous training. This type of analysis could be routinely used as an integrated tool, for example, in planned department meetings to address critical aspects of operative management that could affect both the efficiency and safety profile of the procedure.

The AI step detection model presented in this study can serve as a foundation for many other potential applications. In 2019, Bodenstedt et al. combined both visual data from endoscopic video and surgical device data and were able to train an algorithm to predict the remaining operative time in a range of laparoscopic surgical procedures [38]. Regarding surgical workflow, benefits could apply to hospital schedules, as the ability to predict surgical step duration in real-time would allow surgical staff to more accurately assess remaining procedural time [46, 47]. Other aspects of the context-aware operating room are also being investigated. Surgical tool recognition, for example, was successfully applied to videos of laparoscopic cholecystectomy, with an average classification precision of 93.75% [45].

Surgical video analysis has been used for educational purposes, resulting in efficacy that is equivalent and even superior to conventional methods [46–49]. It can improve surgical perception and awareness and shorten the learning curve [50]. This is expressed in the approved definition of digital surgery: “the use of technology for the enhancement of preoperative planning, surgical performance, therapeutic support, or training, to improve outcomes and reduce harm” [9].

Lastly but perhaps most importantly, one of the most promising potential use cases of AI-based surgical intelligence platforms is the ability to provide real-time decision support to operating surgeons. This capability requires further work, as future models will need to recognize additional procedural components, such as intraoperative adverse events, and to provide predictions and guidance with very high accuracy.

This study does have several limitations. While the model performs well when faced with outliers, as described above, the surgical steps defined are rather generalized. Future efforts should focus on the production of additional standardized step detection rules to further minimize the variability and improve the reproducibility of the model. Additionally, the change of focus step might have added confusion specific to TEP, as compared to other procedures. The fact that the dataset was composed of both bilateral and unilateral procedures, which differ in workflow, may have reduced the model’s accuracy. Moreover, the videos included in our dataset were derived from different venues and operating surgeons in unequal proportions. Namely, 74.5% of videos were collected from a single medical center, possibly contributing to data bias in terms of surgical techniques.

## Conclusions

This study demonstrates that an AI model can provide fully automated TEP video step detection with high accuracy. Accurate step detection constitutes the first step for gaining video-based insights into the TEP procedure and may eventually enable its standardization and facilitate the prediction and prevention of negative outcomes. Accurate step detection is an important capability in itself, allowing videos to be navigated easily and operative ongoings to be presented in a simpler manner. It also enables extraction of efficiency and quality metrics based on step duration, and more. This capability paves the way for further important use cases in surgical training, surgeon self-assessment, performance review, and quality improvement. Future applications include predicting remaining operative time in any given procedure as well as automating surgical reporting [39]. As machine learning continues to advance rapidly, capabilities will evolve from retrospective analyses to online or real-time analyses, with the holy grail being data-driven intraoperative decision support.
